# Evaluation and comparison of therapeutic effects of probiotics and colloidal bismuth subcitrate on abdominal bloating 

**DOI:** 10.22088/cjim.14.3.518

**Published:** 2023

**Authors:** Maryam Soheilipour, Elham Tabesh, Soheila Najmi, Mostafa Raisi, Peyman Adibi

**Affiliations:** 1 Isfahan Gastroenterology and Hepatology Research Center (IGHRC), Isfahan University of Medical Sciences, Isfahan, Iran; 2 Department of Internal Medicine, Isfahan University of Medical Sciences, Isfahan, Iran; 3 Poursina Hakim Digestive Diseases Research Center, Isfahan University of Medical Sciences, Isfahan, Iran

**Keywords:** Abdominal bloating, Functional gastrointestinal disorder, Probiotic, Bismuth, Clinical trial.

## Abstract

**Background:**

Functional abdominal bloating is one of the functional gastrointestinal disorders (FGIDs). Here, we aimed to investigate and compare the effects of probiotics and colloidal bismuth subcitrate on abdominal bloating.

**Methods:**

This was a double-blinded randomized clinical trial performed on 125 patients with functional abdominal bloating in Isfahan in 2020-2021. At the beginning of the study, information on the frequency of abdominal bloating, its severity, the occurrence of early satiety, frequency of borborygmus, frequency of belching, and the frequency of defecation per week was collected. Patients were divided into 3 groups receiving familact probiotic pills that contained 7 bacterial strains, colloidal bismuth subcitrate tablets, and placebo pills for 4 weeks. Afterwards, the frequency and severity of abdominal bloating and other symptoms were compared.

**Results:**

After 2 weeks, patients in the probiotic group had a significantly lower frequency of abdominal bloating compared to other groups (P= 0.006). After 4 and 8 weeks, patients in the probiotic group and bismuth group had a lower frequency of bloating )3.18±3.02, 4.11±3.34) compared to placebo (5.10±3.54) (P= 0.001 and P= 0.037, respectively). During the study, patients in the probiotic group had a significantly lower bloating severity (41.90% had no symptom) compared to bismuth and placebo groups (12.50 ,12.00% had no symptom) (p< 0.05). The frequency of borborygmus was significantly lower in the probiotic group after 2 and 4 weeks during the study compared to other groups (1.62±0.2 Vs 2.69±0.3, 2.45±0.3 ) (P= 0.010 and P= 0.013, respectively).

**Conclusion:**

According to our data, consumption of probiotics improves the frequency and severity of abdominal bloating and reduces borborygmus. Colloidal bismuth subcitrate also has significant effects.

Abdominal bloating usually results from a buildup of gas in the gastrointestinal tract or fluid retention in the abdomen. Abdominal bloating is the accumulation of gas in the gastrointestinal tract, characterized by flatulence and the presence of pressure in the abdomen after eating and belching (1). Abdominal bloating can be the result of normal bodily processes or stems from a condition that affects the digestive system (2). Two main effective factors result in abdominal bloating. One is swallowing air during eating, and the other is gas originating from the breakdown of undigested food. In the first case, swallowing air causes gas to accumulate in the stomach and can occur due to eating too fast or not chewing properly (3). In the second case, the body does not digest some sugars in the small intestine, and these substances react with bacteria in the large intestine and produce gas (4).

Abdominal bloating is one of the most common gastrointestinal complaints in people, indicating that about 6–13% of the general population in Western countries suffer from abdominal distention due to abdominal bloating (5,6). Other common causes of abdominal bloating and abdominal distention in patients include consumption of high-calorie and high-fat meals, rapid consumption of food that reduces the speed of movement and excretion of gas and its retention (7,8). Accumulation of gas due to abdominal bloating can cause the abdomen to dilate and stretch, pushing the diaphragm upwards and reducing the expansion of the lungs (9). On the other hand, there is no pathological or structural cause for abdominal bloating in many patients. In other words, abdominal bloating is often considered a functional disease (10). 

Different treatments such as prescribing chemical drugs and dietary changes (reducing the consumption of legumes and eggs and increasing the consumption of fruits and vegetables) are taken to improve people with bloating (1,11). Abdominal bloating has also been shown to occur in patients with other gastrointestinal diseases, such as irritable bowel syndrome (IBS) (4). Various studies have used drug treatments for abdominal bloating to date, including activated charcoal, bismuth sub-salicylate, alpha-galactosidase, simethicone, and probiotics (12,13). 

Colloidal bismuth subcitrate, is taken to treat temporary discomforts of the stomach and gastrointestinal tract, including nausea, heartburn, indigestion, and diarrhea (14). The use of bismuth in previous studies has had a positive effect on reducing abdominal bloating (15,16). The justification for using probiotics in abdominal bloating is the loss of the normal intestinal flora and gastrointestinal tract. Probiotics contain beneficial bacteria that can inhibit the growth and proliferation of other harmful bacteria through their growth and activity (17). They can also contribute to the body’s health by synthesizing some essential nutrients such as amino acids and vitamins (18). Previous studies used probiotics containing lactobacillus and bifidobacterium, indicating a positive effect on reducing abdominal bloating (19,20). Therefore, given the problems caused by bloating for patients and previous studies examining the effect of bismuth and probiotics in the treatment of patients, this study aimed to evaluate and compare the effect of these two drugs in reducing abdominal bloating in patients.

## Methods

This is a double-blinded randomized clinical trial performed in Khorshid Hospital affiliated with Isfahan University of Medical Sciences in 2020-2021. The current study was conducted on patients with functional abdominal bloating, referred to the gastroenterology clinic of Khorshid hospital. The Research Committee of Isfahan University of Medical Sciences approved the study protocol, and the ethics committee confirmed it (Ethics code: IR.MUI.MED.REC.1399.193, Iranian registry of clinical trials (IRCT) code: IRCT20200601047621N1).

The inclusion criteria were age between 20 to 65 years, diagnosis of abdominal functional bloating by an expert gastroenterologist according to ROME IV criteria, presence of abdominal bloating at least for 3 days a week for the past 3 months, the first experience of abdominal functional bloating for at least 6 months before the study, and signing the written informed consent to participate in this study. Patients with any history of chronic inflammatory bowel disease or structural disease of the gastrointestinal tract, any serious physical problems or illnesses with inflammation or malignancy basis, use of calcium channel blockers in the last 3 months, a severe stressful condition in the last 6 months, positive family history for colon cancer, lactase deficiency disease, celiac disease, treatments with any antibiotics or probiotics 8 weeks before the study, and pregnancy or breastfeeding did not enter the study. The exclusion criteria were the tendency to leave the study during treatment and lack of proper cooperation and drug compliance. Patients were also asked not to use any other drugs during the study course. Otherwise, they were excluded. These drugs included therapeutic agents for diabetes, lung disease, herbal medicine, PPIs, and prokinetics.

To reach 95% confidence level and 80% of test power, 54 patients in each group were considered. These groups were probiotics, bismuth and placebo groups. At the beginning of the study, the patients’ demographic information (including age and gender) was collected by a checklist. We asked the frequency of abdominal bloating per week in patients while measuring the severity of bloating by a 4-item questionnaire as follows: A score of 1 or mild bloating indicated bloating that did not cause problems in the patient's daily activities and was easily tolerated by the patient. A score 4 showed severe bloating that required immediate medical intervention. Previous studies assessed and confirmed the validity and reliability of this scoring system (21). According to bloating scoring system, the score of 1 is considered as a tolerable bloating with no interferes in patient’s daily activities. Score of 2 is considered as bloating that interferes some of patient’s daily activities such as sleeping but requires no treatments. Score 3 is a bloating that interacts with patient’s activities and require medical treatments and score 4 is considered as severe bloating that requires immediate medical treatments. It should be noted that bloating was diagnosed by clinical criteria and gastrointestinal endoscopy was performed only in cases with alarm signs. The diagnosis of bloating was made according to ROME IV criteria. Furthermore, we collected information regarding the occurrence of early satiety per week, frequency of borborygmus per week, frequency of belching per week, and also the frequency of defecation per week among patients at the beginning of the study. Afterwards, the patients were divided into 3 groups, the first of which received familact probiotic pills (Zist Takhmir Company) containing 7 bacterial strains (*Lactobacillus rhamnosus*, *Lactobacillus casei*, *Lactobacillus bulgaricus*, *Lactobacillus acidophilus*, *Bifidobacterium breve*, *Bifidobacterium longum*, *Streptococcus thermophilus* (109 CFU), and fructooligosaccharides). 

The patients received these pills every 12 hours and after the main meals. The second group received colloidal bismuth subcitrate tablets at a dose of 120 mg every 12 hours after meals, and the third group received placebo pills containing glucose every 12 hours. The duration of treatment was 4 weeks. Treatments lasted for 4 weeks, and none of the patients, clinical assessors, and data analyzers were aware of the patients' groupings and the pills. All patients were visited and examined 4 times during the study, including before and 2, 4, and 8 weeks after study. We measured and evaluated the frequency and severity of abdominal bloating and other symptoms. The obtained data were entered into the Statistical Package for Social Sciences (SPSS) version 24. Quantitative data were reported as mean± standard deviation and qualitative data as frequency distribution (percentage). One-way ANOVA or Kruskal Wallis test with Bonferroni test as post hoc, Chi-square test, Fisher’s test, and multivariate repeated measurements were employed to analyze the data. A p-value< 0.05 was considered as a significance threshold.

## Results

**Figure 1:**
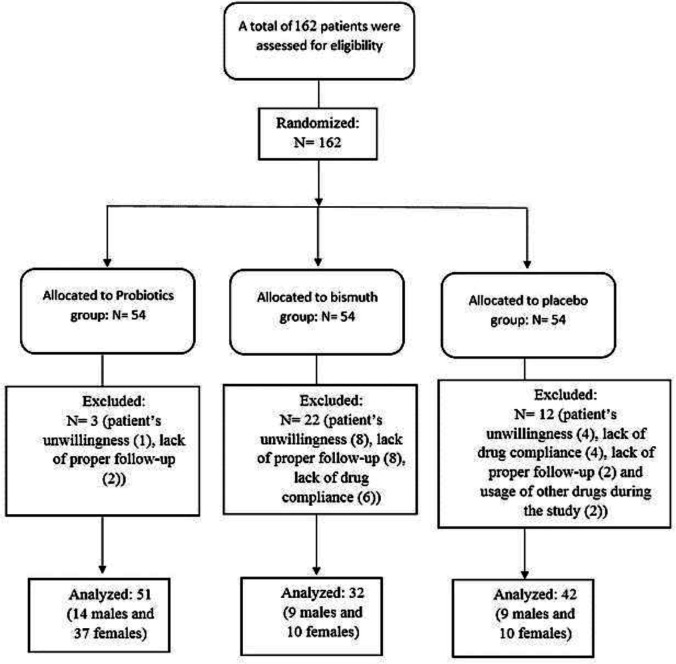
The CONSORT flow diagram of the patients

The present study assessed 162 patients in three groups. During the study, 37 patients were excluded due to their unwillingness (N= 13), lack of drug compliance (N= 10), lack of proper follow-up (N= 12), and taking other drugs during the study (N= 2), leading to the data of 125 patients for analysis. Figure 1 indicates the CONSORT flow chart of the study. The study population consisted of 38 (29.7%) males and 87 (70.3%) females with a mean age of 44.29± 12.39 years. Initial analysis of demographic data showed no significant differences between the three groups regarding age (P= 0.488) and gender (P= 0.052) (table 1). 

**Table 1:** Evaluation of demographic data of patients

		**Probiotic** **(N= 51)**	**Placebo** **(N= 42)**	**Colloidal bismuth subcitrate (N= 32)**	**P-value**
**Sex**	**female**	37 (42.5%)	17 (40.4%)	23 (69.7%)	0.052
	**male**	14 (36.8%)	15 (59.6%)	9 (30.3%)	
**age**		43.25± 12.36	47.18± 12.69	42.10± 12.82	0.488

The three groups had no differences in time frequency of abdominal bloating based on ROME IV questionnaire and its severity at the beginning of the study (P= 0.452 and P= 0.372, respectively). Within 2 weeks, patients in the probiotic group had a significantly lower frequency of abdominal bloating compared to other groups (P= 0.006). Within 4 and 8 weeks, patients in the probiotic and bismuth groups had a lower frequency of bloating compared to placebo (P= 0.001 and P= 0.037, respectively). During the study, patients in the probiotic and bismuth groups had significantly lower bloating severity compared to placebo (p< 0.05). 

We should note that the frequency and severity of bloating decreased significantly in patients that received bismuth and probiotic over the study period (p< 0.05). Table 2 indicates these data. Evaluation of occurrence of early satiety, frequency of belching, and the frequency of defecation per week among groups indicated no significant differences between them during the study (p> 0.05). Only the frequency of borborygmus was significantly lower in the probiotic group after 2 and 4 weeks compared to other groups (P= 0.010 and P= 0.013, respectively). However, there were no significant differences between bismuth and placebo groups (p> 0.05), but we observed significant improvements in placebo and bismuth groups during the study regarding early satiety, borborygmus, and defecation (p< 0.05), and belching showed improvements only in the probiotic group (P= 0.006) (table 3). 

Using repeated measure test, we observed significantly better conditions in the probiotic group regarding the number and severity of abdominal bloating compared to the bismuth and placebo group (p< 0.001) and significantly lower borborygmus compared to placebo (P= 0.027). Patients treated with bismuth showed significant improvements in the number and severity of bloating compared to placebo group (p< 0.05). 

**Table 2:** Evaluation and comparison of bloating severity and frequency among groups

		**Probiotic** **(N= 51)**	**Placebo** **(N= 42)**	**Colloidal bismuth subcitrate (N= 32)**	**P-value** ^1^
**Bloating frequency 0 week**		7.23±2.21*	7.14±4.52	7.33±3.14	0.452
**Bloating frequency 2 weeks**		3.63±3.17	5.69±3.32	5.28±3.61	0.006
**Bloating frequency 4 weeks**		3.18±3.02	5.10±3.54	4.11±3.34	0.001
**Bloating frequency 8 weeks**		4.71±3.08	6.62±2.08	4.17±2.62	0.037
**P-value** ^2^		0.000 ^a^	0.231 ^b^	0.000 ^c^	
**Bloating severity 0**	**No symptom**	0 (0.00)	0 (0.00)	0 (0.00)	0.372^3^
	**Grade 1**	6 (11.80)	4 (12.50)	3 (7.10)	
	**Grade 2**	18 (35.30)	8 (25.00)	10 (23.80)	
	**Grade 3**	25 (49.00)	20 (62.50)	28 (66.70)	
	**Grade 4**	2 (3.90)	0 (0.00)	1 (2.40)	
**Bloating severity 2**	**No symptom**	13 (28.90)	1 (3.80)	3 (7.50)	0.000
	**Grade 1**	12 (26.70)	5 (19.20)	6 (15.00)	
	**Grade 2**	16 (35.60)	7 (26.90)	21 (52.50)	
	**Grade 3**	3 (6.70)	13 (50.00)	9 (22.50)	
	**Grade 4**	1 (2.20)	0 (0.00)	1 (2.50)	
**Bloating severity 4**	**No symptom**	18 (41.90)	3 (12.00)	5 (12.50)	0.000
	**Grade 1**	11 (25.60)	6 (24.00)	12 (30.00)	
	**Grade 2**	13 (30.20)	6 (24.00)	16 (40.00)	
	**Grade 3**	0 (0.00)	10 (40.00)	6 (15.00)	
	**Grade 4**	1 (2.30)	0 (0.00)	1 (2.50)	
**Bloating severity 8**	**No symptom**	7 (17.90)	1 (5.30)	2 (5.70)	0.006
	**Grade 1**	11 (28.20)	2 (10.50)	13 (37.10)	
	**Grade 2**	17 (43.60)	6 (31.60)	13 (37.10)	
	**Grade 3**	3 (7.70)	10 (52.60)	6 (17.10)	
	**Grade 4**	1 (2.60)	0 (0.00)	1 (2.90)	
**P-value**		0.000 ^a^	0.042 ^b^	0.000 ^c^	

* Data are represented by mean ± SD or N (column %); P-value1: Kruskal Wallis test; P= 2: Friedman test; P=3: Chi-square test; different letters (a-c) show significant statistical difference in between group repeated measurements analysis.

**Table 3:** Comparison of early satiety, frequency of borborygmus, frequency of belching, and frequency of defecation among groups

	**Probiotic (N= 51)**	**Placebo (N= 42)**	**Colloidal bismuth subcitrate (N= 32)**	**P-value** ^1^
**Early satiety 0**	2.12±0.4	2.41±0.3	3.45±0.4	0.192
**Early satiety 2**	1.21±0.3	1.72±0.2	2.18±0.3	0.792
**Early satiety 4**	1.11±0.2	1.83±0.2	2.01±0.3	0.938
**Early satiety 8**	1.01±0.2	1.91±0.2	1.11±0.2	0.934
**P-value** ^2^	0.014 ^a^	0.125 ^a^	0.022 ^a^	
**Borborygmus 0**	2.55±0.3	2.61±0.3	2.97±0.3	>1
**Borborygmus 2**	1.81±0.2	2.77±0.2	2.86±0.3	0.010
**Borborygmus 4**	1.62±0.2	2.69±0.3	2.45±0.3	0.013
**Borborygmus 8**	1.14±0.2	2.51±0.3	2.16±0.3	0.318
**P-value**	0.001 ^a^	0.687 ^b^	0.001 ^a, b^	
**Belching 0**	2.33±0.3	3.15±0.4	2.21±0.6	0.164
**Belching 2**	1.76±0.2	2.83±0.3	1.74±0.2	0.249
**Belching 4**	1.43±0.2	1.96±0.2	2.33±0.5	0.285
**Belching 8**	1.01±2	1.89±0.2	2.49±0.5	0.471
**P-value**	0.006 ^a^	0.130 ^a^	0.272 ^a^	
**Defecation 0**	6.13±2.9	7.11±5.2	5.32±2.6	0.079
**Defecation 2**	6.54±2.6	6.63±3.7	6.18±3.2	0.700
**Defecation 4**	7.08±3.1	6.28±3.4	6.88±3.6	0.498
**Defecation 8**	7.39±3.1	7.41±3.2	7.64±4.1	0.615
**P-value**	0.001 ^a^	0.356 ^a^	0.003 ^a^	

* Data are represented by mean ± SD; P=1: Fisher test; P-value2: within group repeated measurements; different letters (a-b) show significant statistical difference in between group repeated measurements analysis.

## Discussion

The present study aimed to investigate and compare the therapeutic effects of probiotic pills and colloidal bismuth subcitrate tablets on abdominal bloating, its severity, early satiety, borborygmus, belching, and defecation. As the results of our study indicated, patients in the probiotic group had a significantly lower frequency of abdominal bloating within 2 weeks, while patients in the probiotic and bismuth groups had a lower frequency of bloating compared to placebo within 4 and 8 weeks. At the end of the study, patients in the probiotic and bismuth groups had significantly lower bloating severity. Besides, the frequency of borborygmus was significantly lower in the probiotic and bismuth groups within 2 and 4 weeks after the study compared to placebo group. Our data demonstrated a significantly better condition in the probiotic group regarding the number and severity of abdominal bloating compared to the bismuth and placebo groups.

These findings support the effectiveness of probiotic pills and bismuth in reducing the patients’ symptoms, but as mentioned above, probiotics had significantly better effects compared to bismuth. Studies have investigated the effects of probiotics on different gastrointestinal issues, including bloating. Yoon et al. (2014) investigated the effects of multispecies probiotics on 49 patients with IBS, indicating that abdominal bloating, the severity of bloating, and abdominal pain improved significantly in patients receiving probiotics (22). Another study by Jafari et al. evaluated the effects of probiotics on bloating and IBS, showing that patients receiving probiotics for 4 weeks had significant improvements in abdominal bloating and pain (23). Dale et al. (2019) also conducted a review study on this issue. It was discussed that the administration of probiotics could result in significant improvements in abdominal bloating and its severity, but a reasonable duration of treatments was required to reach this goal (24). The results of our study were in line with these findings. We showed that the administration of probiotic pills resulted in significant improvements of abdominal bloating within 2 weeks, and the frequency of borborygmus was significantly lower in the probiotic group within 2 and 4 weeks after the study compared to other groups. 

These data support the use of probiotics in patients with abdominal bloating. Recently, Wagner et al. (2021) have indicated that patients receiving probiotics had significantly lower rates of bloating (25). Agah et al. (2020) performed a systematic review with meta-analysis, investigating the effects of probiotics on abdominal function. Based on this study, probiotics were significantly useful in reducing the frequency and severity of bloating, also affecting other symptoms, including borborygmus significantly (26). The results of our study were in line with these findings. However, others have declared that despite the effectiveness of this medication, further investigations and data are still required (27) .Recent studies have demonstrated that normalizing the bacterial flora of the gastrointestinal tract and changes in the absorption procedures mediate the effects of probiotic pills on abdominal symptoms and their severity, and as a result, probiotics could be an effective option in these patients. We also showed that colloidal bismuth subcitrate had significant effects in improving the frequency and severity of abdominal bloating, also shown in previous studies. It has been declared that colloidal bismuth subcitrate could impact the helicobacter pylori and gastric complaints and might reduce other abdominal complications, including bloating (28, 29), but there is still a lack of sufficient data. Based on the findings of our study, administration of colloidal bismuth subcitrate resulted in a significant reduction in bloating frequency and severity and also improvements in the borborygmus and defecation in patients. 

These findings indicate that both probiotic pills and colloidal bismuth subcitrate improved abdominal bloating significantly, and physicians should pay more attention to these properties of the two drugs. The limitations of our study included restrictions in the study population and failure to evaluate other associated factors such as quality of life in patients. However, we observed significant results in this study, believing that probiotics and colloidal bismuth subcitrate could have significant clinical applications. Administration of probiotic pills and colloidal bismuth subcitrate resulted in significant improvements in the frequency of abdominal bloating, its severity, and borborygmus. However, the effects of probiotics started earlier than colloidal bismuth subcitrate in clinical practice. We also suggest further evaluations on larger populations.

## References

[1] Lacy BE, Cangemi D, Vazquez-Roque M (2021). Management of chronic abdominal distension and bloating. Clin Gastroenterol Hepatol.

[2] Mari A, Backer FA, Mahamid M (2019). Bloating and abdominal distension: clinical approach and management. Adv Ther.

[3] Malagelada JR, Accarino A, Azpiroz F (2017). Bloating and abdominal distension: old misconceptions and current knowledge. Am J Gastroenterol.

[4] Kanazawa M, Miwa H, Nakagawa A (2016). Abdominal bloating is the most bothersome symptom in irritable bowel syndrome with constipation (IBS-C): a large population-based internet survey in Japan. Biopsychosoc Med.

[5] Schmulson MJ, Chiu-Ugalde J, Sáez-Ríos A (2020). Efficacy of the combination of pinaverium bromide 100 mg plus simethicone 300 mg in abdominal pain and bloating in irritable bowel syndrome: a randomized, placebo-controlled trial. J Clin Gastroenterol.

[6] Defrees DN, Bailey J (2017). Irritable bowel syndrome: epidemiology, pathophysiology, diagnosis, and treatment. Primary Care.

[7] DiNicolantonio JJ, Lucan SC (2015). Is fructose malabsorption a cause of irritable bowel syndrome?. Med Hypotheses.

[8] Rao SS, Rehman A, Yu S, De Andino NM (2018). Brain fogginess, gas and bloating: a link between SIBO, probiotics and metabolic acidosis. Clin Transl Gastroenterol.

[9] Wilkinson JM, Cozine EW, Loftus CG (2019). Gas, bloating, and belching: approach to evaluation and management. Am Fam Physian.

[10] Kamboj AK, Oxentenko AS (2018). Workup and management of bloating. Clin Gastroenterol Hepatol.

[11] Kwiatkowski L, Rice E, Langland J (2017). Integrative Treatment of Chronic Abdominal Bloating and Pain Associated With Overgrowth of Small Intestinal Bacteria: A Case Report. Altern Ther Health Med.

[12] Nelson AD, Black CJ, Houghton LA, Lugo‐Fagundo NS, Lacy BE, Ford AC (2021). Systematic review and network meta‐analysis: efficacy of licensed drugs for abdominal bloating in irritable bowel syndrome with constipation. Aliment Pharmacol Ther.

[13] Babak A, Rouzbahani R, Khalili Nejad R, Rafiee Zadeh A (2019). Comparison of nutritional behaviors and physical activities between overweight/obese and normal-weight adults. Adv Biomed Res.

[14] Vega-Jiménez A, Almaguer-Flores A, Flores-Castañeda M (2017). Bismuth subsalicylate nanoparticles with anaerobic antibacterial activity for dental applications. Nanotechnology.

[15] Daghaghzadeh H, Memar A, Mohamadi Y (2018). Therapeutic effects of low-dose bismuth subcitrate on symptoms and health-related quality of life in adult patients with irritable bowel syndrome: a clinical trial. J Res Pharm Practice.

[16] Alavinejad P, Hashemi SJ, Hajiani E (2016). Low dose mesalazine plus bismuth regimen and symptoms of irritable bowel syndrome in patients with bloating: a quasi-experimental study. Jundishapur J Chronic Dis Care.

[17] Ford AC, Harris LA, Lacy BE, Quigley EMM, Moayyedi P (2018). Systematic review with meta‐analysis: the efficacy of prebiotics, probiotics, synbiotics and antibiotics in irritable bowel syndrome. Aliment Pharmacol Ther.

[18] Hungin A, Mitchell C, Whorwell P (2018). Systematic review: probiotics in the management of lower gastrointestinal symptoms–an updated evidence‐based international consensus. Aliment Pharmacol Ther.

[19] Vitellio P, Celano G, Bonfrate L (2019). Effects of Bifidobacterium longum and Lactobacillus rhamnosus on gut microbiota in patients with lactose intolerance and persisting functional gastrointestinal symptoms: A randomised, double-blind, cross-over study. Nutrients.

[20] Bonfrate L, Di Palo DM, Celano G (2020). Effects of bifidobacterium longum bb536 and lactobacillus rhamnosus hn001 in ibs patients. Eur J Clin Investigat.

[21] Schmulson M, Chang L (2011). The treatment of functional abdominal bloating and distension. Aliment Pharmacol Ther.

[22] Yoon JS, Sohn W, Lee OY (2014). lEffect of multispecies probiotics on irritable bowel syndrome: a randomized, double‐blind, placebo‐controlled tria. J Gastroenterol Hepatol.

[23] Jafari E, Vahedi H, Merat S, Momtahen S, Riahi A (2014). Therapeutic effects, tolerability and safety of a multi-strain probiotic in Iranian adults with irritable bowel syndrome and bloating. Arch Iranian Med.

[24] Dale HF, Rasmussen SH, Asiller ÖÖ, Lied GA (2019). Probiotics in irritable bowel syndrome: an up-to-date systematic review. Nutrients.

[25] Wagner NRF, Ramos MRZ, de Oliveira Carlos L (2021). Effects of probiotics supplementation on gastrointestinal symptoms and SIBO after Roux-en-Y gastric bypass: a prospective, randomized, double-blind, placebo-controlled trial. Obes Surg.

[26] Agah S, Akbari A, Heshmati J (2020). Systematic review with meta-analysis: Effects of probiotic supplementation on symptoms in functional dyspepsia. J Functional Foods.

[27] Asha MZ, Khalil SF (2020). Efficacy and safety of probiotics, prebiotics and synbiotics in the treatment of irritable bowel syndrome: a systematic review and meta-analysis. Sultan Qaboos Univ Med J.

[28] Koulinska I, Riester K, Chalkias S, Edwards MR (2018). Effect of bismuth subsalicylate on gastrointestinal tolerability in healthy volunteers receiving oral delayed-release dimethyl fumarate: PREVENT, a randomized, multicenter, double-blind, placebo-controlled study. Clin Ther.

[29] Syam AF, Simadibrata M, Makmun D (2017). National consensus on management of dyspepsia and Helicobacter pylori infection. Acta Medica Indonesiana.

